# TM6SF2 and MAC30, new enzyme homologs in sterol metabolism and common metabolic disease

**DOI:** 10.3389/fgene.2014.00439

**Published:** 2014-12-11

**Authors:** Luis Sanchez-Pulido, Chris P. Ponting

**Affiliations:** Medical Research Council Functional Genomics Unit, Department of Physiology, Anatomy and Genetics, University of OxfordOxford, UK

**Keywords:** TM6SF2, MAC30, TMEM97, EBP, NAFLD, CDPX2, cancer, cholesterol

## Abstract

Carriers of the Glu167Lys coding variant in the TM6SF2 gene have recently been identified as being more susceptible to non-alcoholic fatty liver disease (NAFLD), yet exhibit lower levels of circulating lipids and hence are protected against cardiovascular disease. Despite the physiological importance of these observations, the molecular function of TM6SF2 remains unknown, and no sequence similarity with functionally characterized proteins has been identified. In order to trace its evolutionary history and to identify functional domains, we embarked on a computational protein sequence analysis of TM6SF2. We identified a new domain, the EXPERA domain, which is conserved among TM6SF, MAC30/TMEM97 and EBP (D8, D7 sterol isomerase) protein families. EBP mutations are the cause of chondrodysplasia punctata 2 X-linked dominant (CDPX2), also known as Conradi-Hünermann-Happle syndrome, a defective cholesterol biosynthesis disorder. Our analysis of evolutionary conservation among EXPERA domain-containing families and the previously suggested catalytic mechanism for the EBP enzyme, indicate that TM6SF and MAC30/TMEM97 families are both highly likely to possess, as for the EBP family, catalytic activity as sterol isomerases. This unexpected prediction of enzymatic functions for TM6SF and MAC30/TMEM97 is important because it now permits detailed experiments to investigate the function of these key proteins in various human pathologies, from cardiovascular disease to cancer.

## Introduction

Exome resequencing studies have shown great success in identifying variants that cause rare Mendelian disease (Bamshad et al., 2011). More recently, exome association studies have begun to reveal coding variants that contribute to complex disease risk (Do et al., 2012; Kiezun et al., 2012). To fully understand disease pathoetiology the identification of these variants should be followed by experimental studies that seek to reveal their effects on biochemical pathways and cellular processes. This can be straightforward when much is already known about the mutated gene and its encoded protein. Nevertheless, disease-associated coding variants often lie within sequence or genes that are devoid of annotated functions or features, such as currently defined motifs or domains (Gollery et al., 2006), or they occur within human genes whose proteins have not yet been experimentally characterized. Most human proteins currently have no well-defined molecular function (Lee et al., 2007). Even when proteins contain features such as catalytic amino acids that are indicative of enzymatic activity their substrates may remain unknown (Bartlett et al., 2003; Galperin and Koonin, 2004; Addou et al., 2009). A productive approach to determining the molecular functions of newly-assigned disease genes is to identify, using in-depth protein sequence analyses, homology relationships that reveal evolutionary relationships and domain architectures and, on occasion, explain the molecular and cellular deficits in disease (Goodstadt and Ponting, 2001).

Recently, a coding variant (p.Glu167Lys) in a human gene TM6SF2 (Transmembrane 6 Superfamily Member 2) was found to exceed genome-wide significance for association with total cholesterol and liver fat levels (Dongiovanni et al., 2014; Holmen et al., 2014; Kozlitina et al., 2014; Liu et al., 2014; Sookoian et al., 2014). This amino acid substitution also explains the genome-wide association study signals on chromosome 19p12 for plasma triglyceride or total cholesterol levels, and for increased myocardial infarction risk and non-alcoholic fatty liver disease (NAFLD) susceptibility (Dongiovanni et al., 2014; Holmen et al., 2014; Kozlitina et al., 2014; Mahdessian et al., 2014; Sookoian et al., 2014). These studies showed that TM6SF2 is expressed highly in the liver, with the Glu167Lys variant being expressed at greatly reduced levels, and that alteration of its transcript's levels in the mouse results in changes in liver triglyceride, cholesterol, low-density and high-density lipoprotein levels and in very-low-density lipoprotein (VLDL) secretion.

Nevertheless, the molecular function of TM6SF2 remains unknown, and its only distinguishing features are its predicted 10 transmembrane helices, and its localisation to the endoplasmic reticulum (ER) and the ER-Golgi intermediate compartment in liver cells (Mahdessian et al., 2014). In particular, it is currently unknown whether this protein has value of being a potential drug target (Holmen et al., 2014), and how its variant contributes to liver triglyceride metabolism, coronary artery disease and type 2 diabetes mellitus (Mahdessian et al., 2014). In-depth analysis of protein sequences, so successful previously in explaining the molecular bases of disease (for example, Sanchez-Pulido et al., 2012; Zhang et al., 2012; Babbs et al., 2013), may shed light on its function.

**Figure 1:**
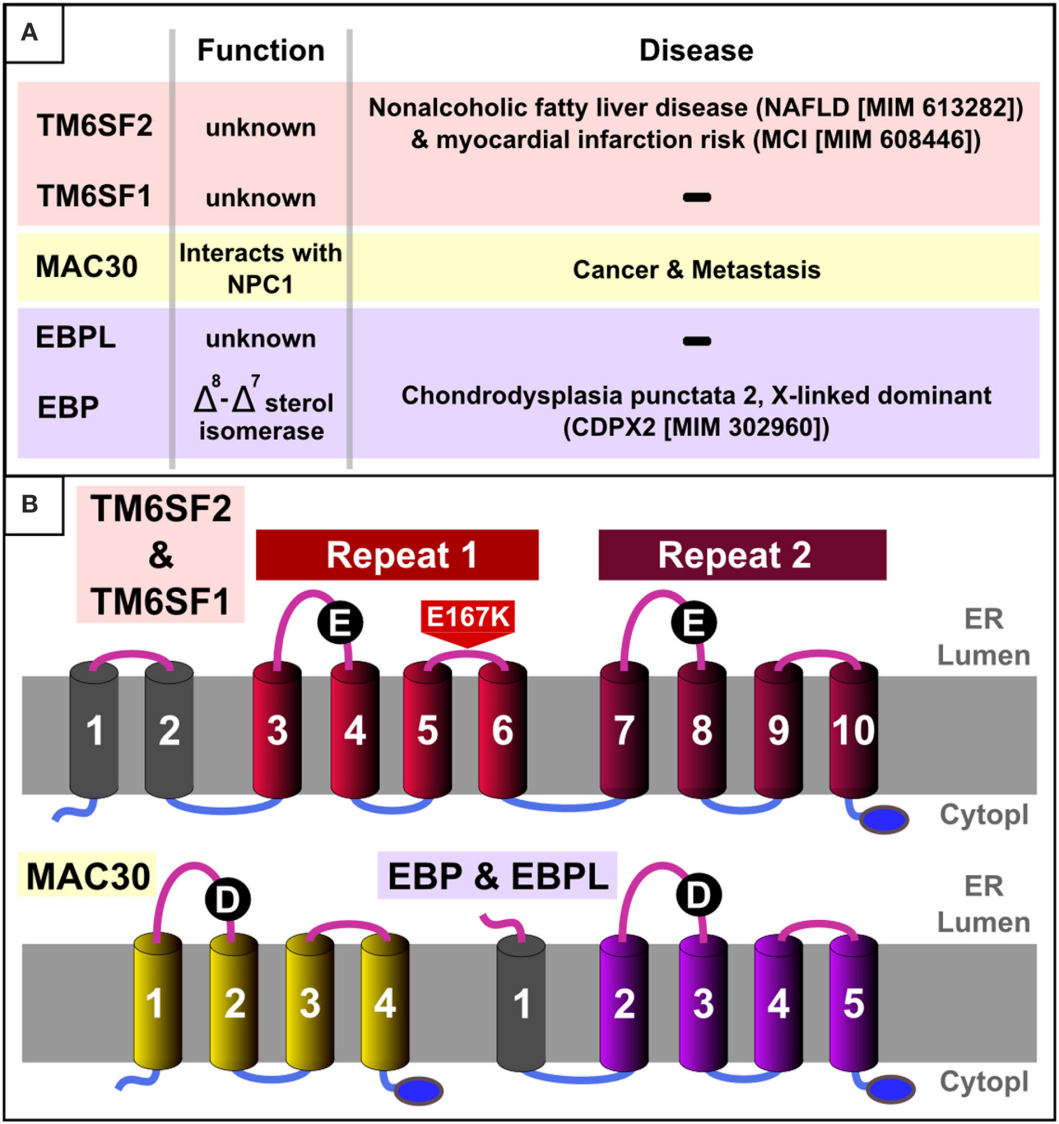
**EXPERA domain-containing proteins in humans. (A)** Summary table of known function/disease relationships for all EXPERA domain-containing proteins in humans. **(B)** Transmembrane topology and common features in human members of the EXPERA domain-containing families. Common topology for the core four transmembrane regions (colored cylinders) present in all members of the EXPERA superfamily (**Figures 3**, S1–S3). The blue oval labels the putative C-terminal ER retention signal (lysine-rich sequence). Black circles label the most conserved position of the EXPERA superfamily (E101 and E255 in TM6SF2, D56 in MAC30 and D108 in EBP).

**Figure 2:**
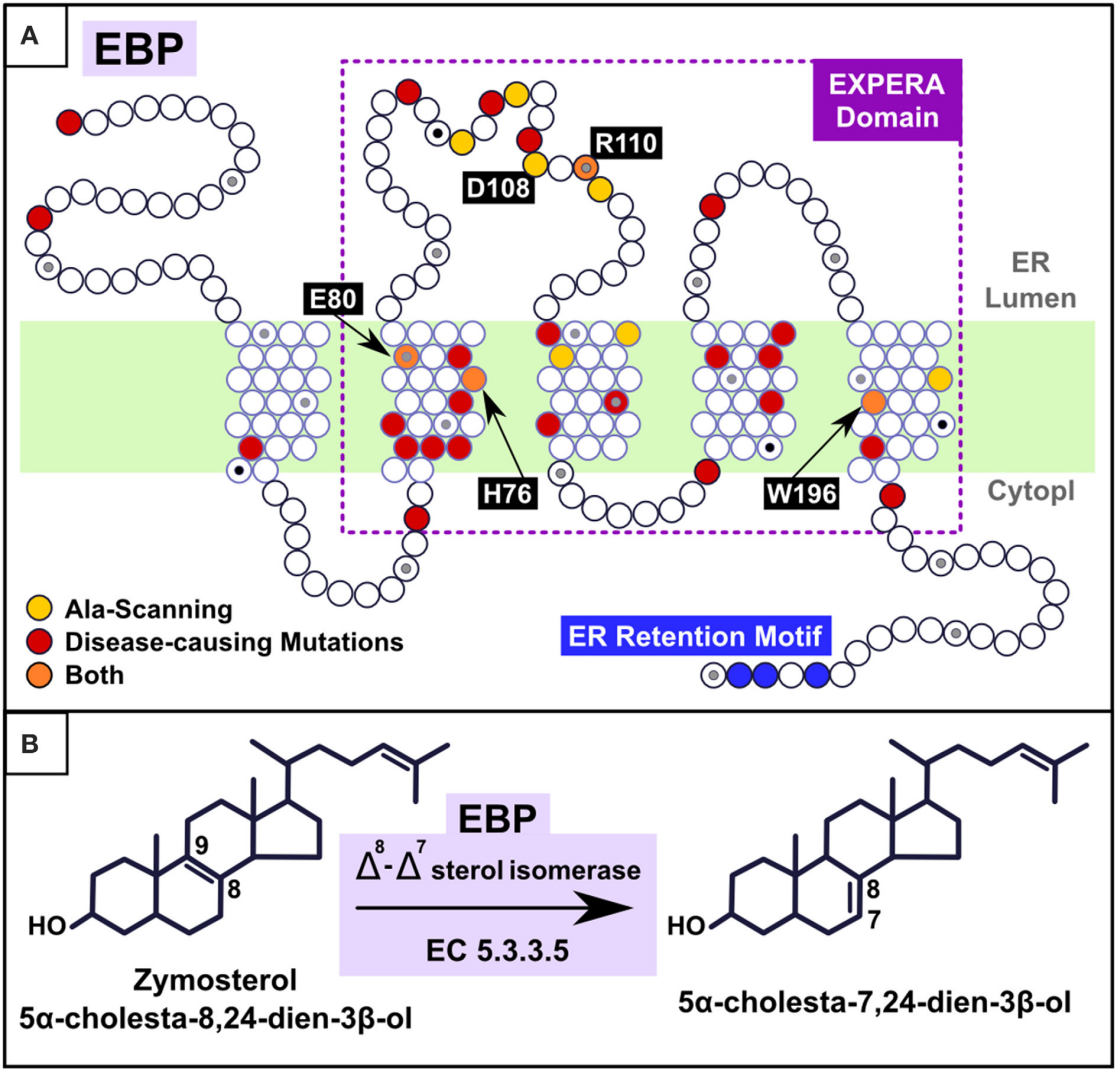
**(A)** Mapping alanine-scanning mutagenesis and known disease causing missense mutations in the EBP model. Alanine-scanning (Moebius et al., 1999) identified 11 residues as major determinants of EBP catalytic activity (His77, Glu81, Trp102, Tyr105, Asp109, Arg111, Tyr112, Glu123, Thr126, Asn194, and Trp197; here renumbered to the current EBP_HUMAN SwissProt entry numbering by subtracting one to the number of each position). Four (orange points) are present in exactly the same positions as are disease associated mutations (H76Y, E80K, R110Q, and W196S) and the remaining seven (W101, Y104, D108, Y111, E122, T125, and N193) (yellow points) are located in the vicinity of disease associated mutations (fewer than five residues-distant). Mapped CDPX2 disease causing missense mutations (red points), derived from Human Gene Mutation Database (HGMD) and PubMed analysis (Stenson et al., 2003), are: M1I (Steijlen et al., 2007), M1V (Hello et al., 2010), R62W (Herman et al., 2002), L66P (Whittock et al., 2003), C67R (Morice-Picard et al., 2011), W68C (Lambrecht et al., 2014), C72Y (Herman et al., 2002), I75N (Barboza-Cerda et al., 2014), H76Y (Umekoji et al., 2008), E80K (Braverman et al., 1999; Ikegawa et al., 2000; Aughton et al., 2003), W82C (Has et al., 2002; Shirahama et al., 2003), S98F (Tysoe et al., 2008), S98P (Tysoe et al., 2008), E103K (Kolb-Mäurer et al., 2008), G107R (Derry et al., 1999), R110Q (Derry et al., 1999; Hou, 2013), V119G (Non-lethal) (Cañueto et al., 2012; Bode et al., 2013), G130V (Herman et al., 2002), S133R (Braverman et al., 1999; Derry et al., 1999), R147G (Becker et al., 2001), R147H (Braverman et al., 1999; Has et al., 2000; Ikegawa et al., 2000; Shirahama et al., 2003), G157S (Herman et al., 2002), D162H (Whittock et al., 2003), L164P (Cañueto et al., 2012), Y165C (Shirahama et al., 2003), G173R (Herman et al., 2002), W196S (Herman et al., 2002), L203P (Has et al., 2002), D206Y (Ausavarat et al., 2008). L18P and W47C (Milunsky et al., 2003; Furtado et al., 2010) present a less severe phenotype called MEND (Male EBP Disorder with Neurological Defects) syndrome (Arnold et al., 2012). **(B)** Reaction catalyzed by EBP. Cholesterol carbon atoms C7, C8, and C9 are label.

**Figure 3:**
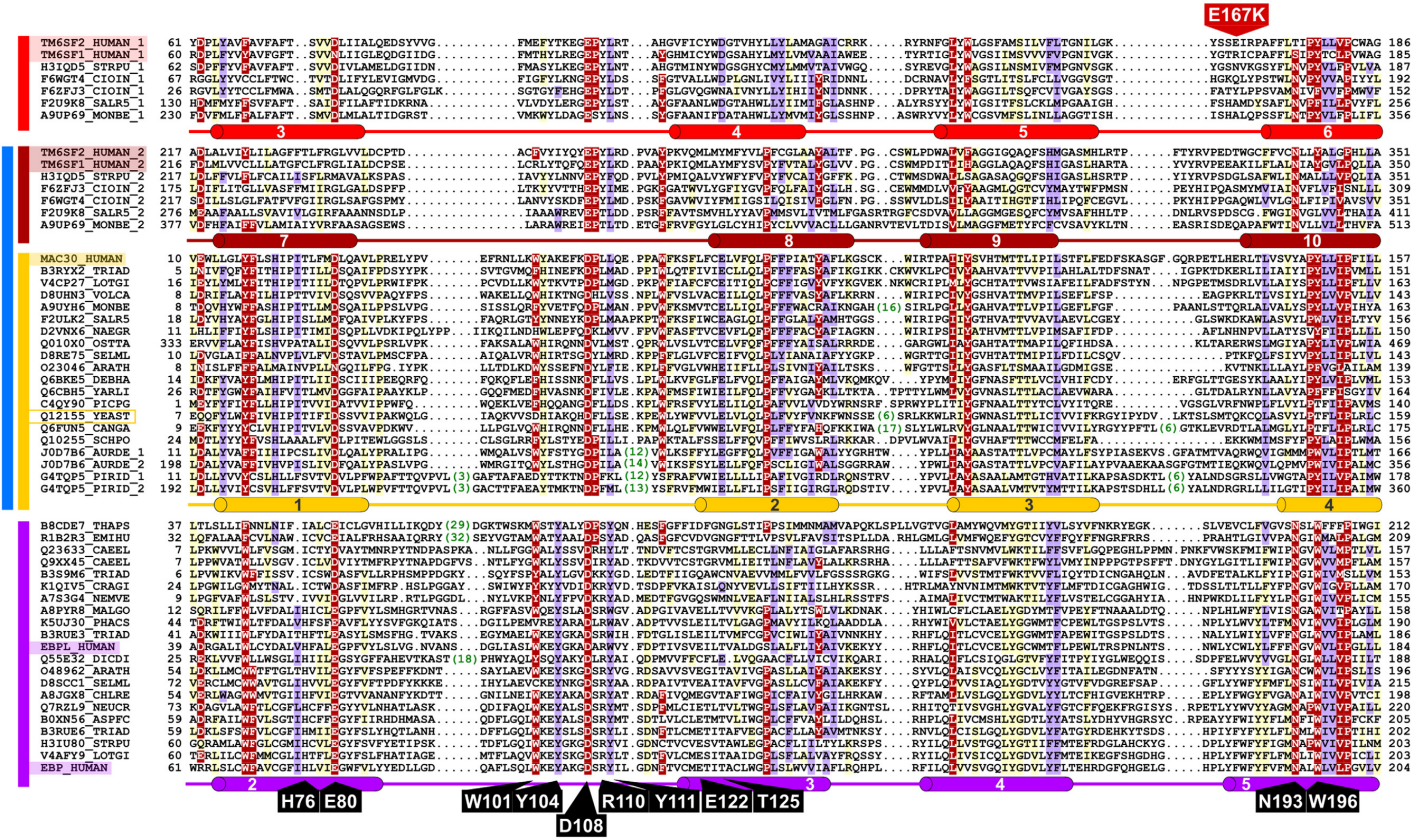
**Representative multiple sequence alignment of the EXPERA domain**. Putative EBP catalytic residues (identified by alanine-scanning) described by Moebius et al. are label in black (Moebius et al., 1999). A mutation identified in TM6SF2 is label in red (Holmen et al., 2014; Kozlitina et al., 2014; Sookoian et al., 2014). Human sequence names are highlighted and the only member of the EXPERA superfamily in *Saccharomyces cerevisiae*, part of the MAC30/TMEM97 family, is indicated by a yellow box. Numbers shown in green represent inserted amino acids that have been removed from the alignment. Different groups of the EXPERA sequences identified by sequence similarity are shown by colored lines to the left of the alignment: light red, TM6SF family first repeat; dark red, TM6SF family second repeat; yellow, MAC30/TMEM97 family; purple, EBP family. DUF2781 (in blue), previously defined in Pfam (includes TM6SF second repeat and MAC30 family). The TMHMM helix transmembrane (Krogh et al., 2001) consensus prediction are shown below the alignment for each family, in red, yellow, and violet cylinders for TM6SF (repeats 1 and 2), MAC30/TMEM97, and EBP families, respectively (see Figures S1–S3). The limits of the protein sequences included in the alignment are indicated by flanking residue positions. Alignments were produced with T-Coffee, HMMer, and HHpred (Eddy, 1996; Notredame et al., 2000; Söding et al., 2005; Finn et al., 2011) using default parameters and slightly refined manually. The alignment was presented with the program Belvu (Sonnhammer and Hollich, 2005) using a coloring scheme indicating the average BLOSUM62 scores (which are correlated with amino acid conservation) of each alignment column: red (>0.7), violet (between 0.7 and 0.4) and light yellow (between 0.4 and 0.2). Sequences are named according to their UniProt identifications (Wu et al., 2006). Species abbreviations: ARATH, *Arabidopsis thaliana* (Mouse-ear cress); ASPFC, *Neosartorya fumigata* (Fungus); AURDE, *Auricularia delicata* (White-rot fungus); CAEEL, *Caenorhabditis elegans*; CANGA, *Candida glabrata* (Yeast); CHLRE, A8JGX8_CHLRE, *Chlamydomonas reinhardtii* (Green alga); CIOIN, *Ciona intestinalis*; CRAGI, *Crassostrea gigas* (Pacific oyster); DEBHA, *Debaryomyces hansenii* (Yeast); DICDI, *Dictyostelium discoideum* (Slime mold); EMIHU, *Emiliania huxleyi* (Chromalveolata); HUMAN, *Homo sapiens*; LOTGI, *Lottia gigantea* (Giant owl limpet); MALGO, *Malassezia globosa* (Fungus); MONBE, *Monosiga brevicollis* (Choanoflagellate); NAEGR, *Naegleria gruberi* (Amoeba); NEMVE, *Nematostella vectensis* (Starlet sea anemone); NEUCR, *Neurospora crassa* (Fungus); OSTTA, *Ostreococcus tauri* (Green alga); PHACS, *Phanerochaete carnosa* (Fungus); PICPG, *Komagataella pastoris* (Yeast); PIRID, *Piriformospora indica* (Fungus); SALR5, *Salpingoeca rosetta* (Choanoflagellate); SCHPO, *Schizosaccharomyces pombe* (Fission yeast); SELML, *Selaginella moellendorffii* (Spikemoss); STRPU, *Strongylocentrotus purpuratus* (Purple sea urchin); THAPS, *Thalassiosira pseudonana* (Marine diatom); TRIAD, *Trichoplax adhaerens*; VOLCA, *Volvox carteri* (Green alga); YARLI, *Yarrowia lipolytica* (Yeast); YEAST, *Saccharomyces cerevisiae* (Baker's yeast).

**Figure 4:**
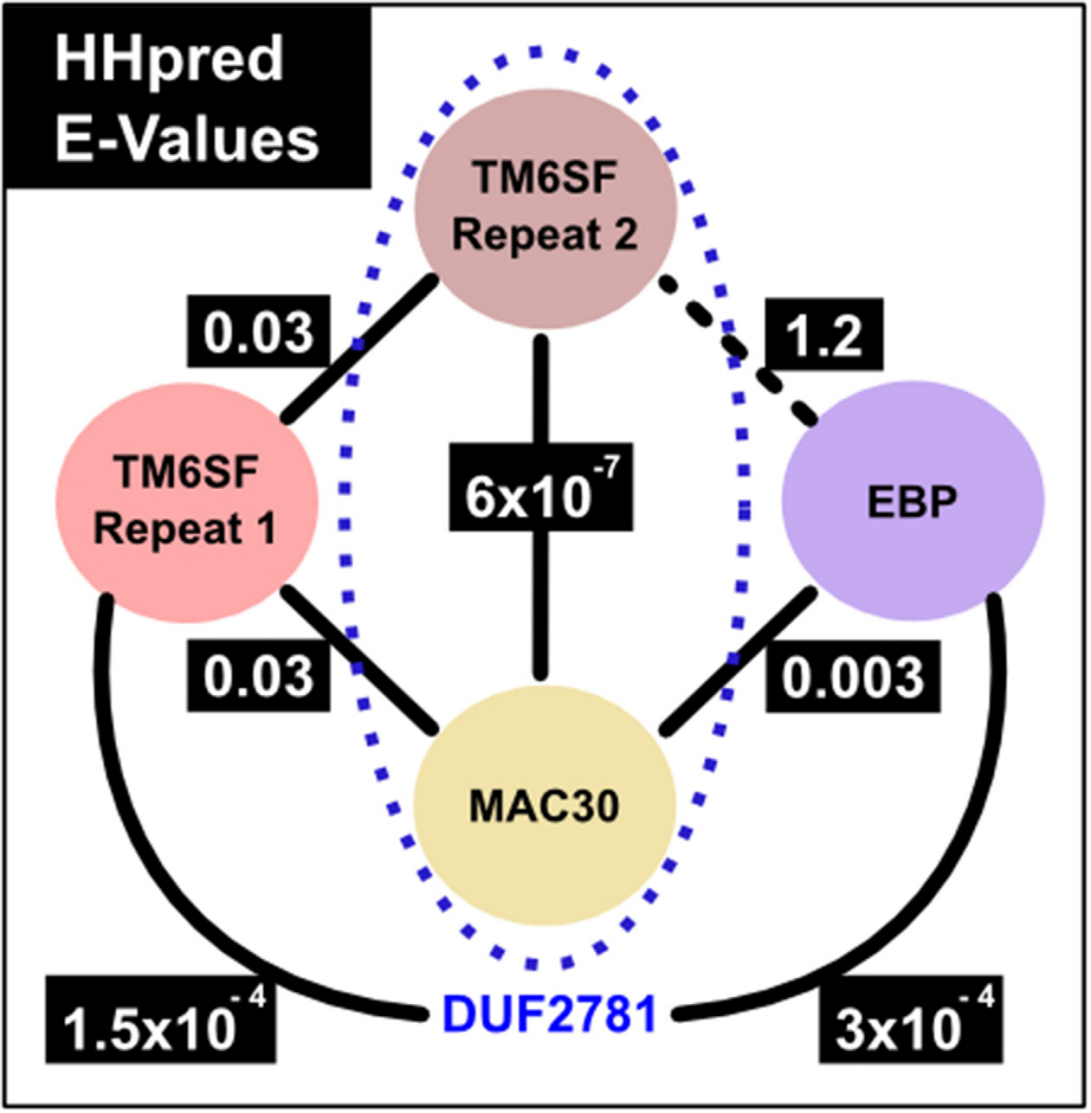
**HHpred comparison E-values**. The numbers correspond to E-values from HHpred (Söding et al., 2005) profile searches against a Pfam profile database which includes profiles that represent families shown in the figure. Profile-to-profile matches were evaluated in terms of an E-value, which is the expected number of non-homologous proteins with a score higher than that obtained for the database match. An E-value much lower than one indicates statistical significance. Solid lines represent statistically significant sequence similarity relationships, e.g., the MAC30/TMEM97 family calibrated profile finds with 0.003 and 0.03 E-values the profiles of EBP family and TM6SF first EXPERA repeat, respectively. The black dotted line between TM6SF second EXPERA domain repeat and EBP shows the unique relationship found with a non-highly significant value (E-value 1.2). The sequence similarity between TM6SF (second EXPERA domain repeat) and MAC30/TMEM97 families (presented inside the blue dotted oval) was already described in a DUF (Domain of Unknown Function) entry of Pfam (DUF2781, Pfam family identification: PF10914) (Bateman et al., 2010; Punta et al., 2012).

Consequently, we embarked on a computational sequence analysis of the TM6SF protein family and identified a novel domain (termed “EXPERA,” see below) that is present twice in TM6SF proteins and that is also conserved among MAC30 (Meningioma-associated protein 30; also known as Transmembrane protein 97 [TMEM97]), and EBP (Emopamil binding protein) protein families (Figures 1–**4**). These observations provide evidence that these previously uncharacterised human proteins have, in common with EBP, an isomerase enzymatic activity contributing to sterol metabolism.

## Material and methods

Multiple sequence alignments for each EXPERA domain-containing families were generated independently with the program T-Coffee using default parameters (Notredame et al., 2000), slightly refined manually and visualized with the Belvu program (Sonnhammer and Hollich, 2005). Profiles of the alignment as global hidden Markov models (HMMs) were generated using HMMer (Eddy, 1996; Finn et al., 2011). Profile-based sequence searches were performed against the Uniref50 protein sequence database (Wu et al., 2006) using HMMsearch (Eddy, 1996; Finn et al., 2011). We used NAIL to view and analyse the HMMsearch results, which provided a formatted view with hyper-links to related web resources and coloring related to taxonomic information, thus facilitating interpretation of the results (Sánchez-Pulido et al., 2000). Remote homology analyses were performed using profile-to-profile comparisons (Söding et al., 2005). The significance of sequence-to-sequence, profile-to-sequence, and profile-to-profile matches were evaluated in terms of an E-value, which reflects the number of observations of better sequence matches expected by chance. Transmembrane predictions were performed using the TMHMM Server (Krogh et al., 2001). Figures were generated using Inkscape (http://inkscape.org/).

## Results and discussion

### Sequence analysis

We started by considering the number of transmembrane regions present in TM6SF protein family members. A few members of a second family, that of MAC30/TMEM97 proteins, are annotated by Pfam as being homologous to TM6SF proteins (Pfam entry: DUF2781—Domain of Unknown Function 2781) (Bateman et al., 2010; Punta et al., 2012). This was puzzling because ten transmembrane regions are consistently predicted for TM6SF proteins (Figures 1, S1) whereas only four such helices are evident for MAC30/TMEM97 proteins (Figures 1, S2). As transmembrane proteins often contain internal duplications (Shimizu et al., 2004), we considered whether TM6SF proteins contain tandem repeats of multiple transmembrane regions. Indeed, using the HHpred profile-profile comparison approach (Söding et al., 2005), and a sequence profile of the last four transmembrane regions of TM6SF (corresponding to human TM6SF2 amino acids 217–351), we identified statistically significant sequence similarity with a profile generated from TM6SF transmembrane regions three to six (corresponding to human TM6SF2 amino acids 61–186; *E* = 0.03). In addition, this approach revealed significant sequence similarity between each of these repeats and a single repeat in the MAC30/TMEM97 family (corresponding to human MAC30/TMEM97 amino acids 10–157; *E* = 6 × 10^−7^ and 0.03; **Figure 4**).

By iteratively improving the phyletic coverage in each protein family using HMMer database searches (Eddy, 1996), we obtained statistical significance from profile-profile comparisons that link these three sequence families (specifically, the two TM6SF repeats and the single MAC30/TMEM97 repeat) to the Emopamil binding protein (EBP) family (Figures 3, 4). The significance of these sequence similarities, their common transmembrane helix configuration, and their shared predicted C-terminal ER retention signal (Figures 1, 2) (Jackson et al., 1990) imply that these domains are homologous, having derived from a common evolutionary ancestor. We name this four transmembrane region the EXPERA (EXPanded EBP superfamily) domain.

### Function prediction

As for the TM6SF family, the molecular function of MAC30/TMEM97 is currently poorly understood. Nevertheless, because of its wide phyletic distribution in eukaryotes (in plants, metazoa and fungi) it is likely to have a fundamental cellular function. As expected from our sequence analysis, it is mainly localized in the ER (Huh et al., 2003; Matsuyama et al., 2006). Under sterol-depleted conditions, however, it becomes enriched in the endo-lysosomal compartment where it interacts with NPC1 (Niemann-Pick disease, type C1 Protein) and regulates cellular cholesterol levels (Wilcox et al., 2007; Bartz et al., 2009). In a variety of cancers, elevated MAC30/TMEM97 expression has been directly related to unfavorable prognosis, and its down-regulation inhibits the proliferation of gastric cancer cells (Kayed et al., 2004; Zhang et al., 2006; Moparthi et al., 2007; Yan et al., 2010; Zhao et al., 2011; Han et al., 2013; Xiao et al., 2013; Yang et al., 2013; Xu et al., 2014).

The only member of the EXPERA superfamily with known molecular function is EBP, an enzyme with a Δ8, Δ7 sterol isomerase activity that catalyzes the transposition of a double bond from C8 = C9 to C7 = C8 in the sterol B-ring (Figure 2) (Wilton et al., 1969; Akhtar et al., 1970; Silve et al., 1996; Bae et al., 2001; Nes et al., 2002; Rahier et al., 2008).

EBP forms homotetramers (Nes et al., 2002) and higher-order protein complexes with sterol Δ7 reductase (DHCR7), catalyzing and regulating key steps in the final cholesterol biosynthesis pathway (Kedjouar et al., 2004; de Medina et al., 2010; Silvente-Poirot and Poirot, 2012). Mutations in EBP cause Conradi-Hünermann-Happle syndrome (also known as Chondrodysplasia punctata type II disease), a rare X-linked dominant disorder characterized by skeletal malformations, skin abnormalities, cataracts, and short stature (Braverman et al., 1999; Derry et al., 1999; Has et al., 2000, 2002; Cañueto et al., 2012, 2014).

Based on consensus transmembrane predictions over the expanded EBP family and superfamily (EXPERA domain-containing proteins) (Figures 3, S3) we generated a five transmembrane EBP model (Figures 1, 2), that adds to and reconciles the main features of previously proposed four transmembrane EBP models (Hanner et al., 1995; Moebius et al., 1997, 2000; Dussossoy et al., 1999). Our model is in agreement with a lumenal localisation of the EBP N-terminal region (Dussossoy et al., 1999), and with a cytoplasmic localisation of its predicted C-terminal ER retention motif (Jackson et al., 1990; Hanner et al., 1995; Moebius et al., 1997, 2000). Mapping alanine-scanning mutagenesis variants (Moebius et al., 1999) and known disease-causing missense mutations (Figure 2) onto the EBP model, predict that the luminal, and transmembrane portions of the EBP EXPERA domain contain the greater fraction of key functional sites (Figures 2, 3).

Several of these sites, previously proposed contain catalytic residues (Moebius et al., 1999), are conserved not just in EBP orthologues but also across the EXPERA domain superfamily (Figure 3). Conservation of acidic amino acids at positions 80 and 108 (human EBP numbering) strongly suggests their involvement in catalysis involving sterols across all members of this superfamily. How these residues might catalyze sterol isomerisation is unclear, but may be similar to the enzymatic action of ketosteroid isomerases for which acidic residues (Asp or Glu) act as a proton donor or acceptor (Pollack, 2004; Sharma et al., 2006). EBP's proposed catalytic mechanism initially involves C-9 protonation of the steroid molecule, with the subsequent generation of a carbonium ion at C-8, and finally the elimination of a proton from C-7 (Wilton et al., 1969; Nes et al., 2002; Rahier et al., 2008) (Figure 1). It is possible that the conserved acidic residues in other EXPERA domain proteins, including MAC30/TMEM97 and TM6SF1/2, catalyze a similar sterol isomerisation reaction as proton donors and acceptors.

The homologous relationships described here between TM6SF2 and EBP could also explain the reported side-effects of tamoxifen (Oien et al., 1999; Hackshaw et al., 2011), which is an antagonist of the estrogen receptor commonly used in breast cancer therapy (Jordan, 2000). Drug cross-reactivity among homologous proteins frequently underlies undesired pleiotropic effects (Searls, 2003; Campillos et al., 2008).

Tamoxifen is a known inhibitor of EBP (Moebius et al., 1998; Kedjouar et al., 2004; de Medina et al., 2010; Silvente-Poirot and Poirot, 2012), and if its homolog TM6SF is similarly inhibited by tamoxifen then this would result in a reduction of total cholesterol level and the induction of NAFLD (Holmen et al., 2014; Kozlitina et al., 2014; Liu et al., 2014; Sookoian et al., 2014) which are precisely the known side-effects of tamoxifen administration (Oien et al., 1999; Hackshaw et al., 2011).

MAC30/TMEM97 is expressed at high levels in breast, esophagus, stomach, and colon cancers (Kayed et al., 2004). Human gastric cancer cells are known to have reduced cellular proliferation and mobility when MAC30/TMEM97 transcript levels are down-regulated (Xu et al., 2014). This implies that the inhibition of MAC30/TMEM97 catalytic activity by tamoxifen may also lead to reduced proliferation of cancer cells. Identification of the EXPERA domain family may thus help to elucidate the complex interplay between cancer and cholesterol metabolism (Silvente-Poirot and Poirot, 2014).

In summary, our analyses have identified TM6SF1, TM6SF2, and MAC30/TMEM97 as EBP homologs. This indicates that these proteins are all likely to possess similar catalytic activities, potentially as sterol isomerases. These results provide new opportunities for their experimental characterization, and for the development of drugs that would inhibit members of the EXPERA superfamily.

## Author contributions

Luis Sanchez-Pulido and Chris P. Ponting designed the research and wrote the paper.

### Conflict of interest statement

The authors declare that the research was conducted in the absence of any commercial or financial relationships that could be construed as a potential conflict of interest.

## Supplementary material

We include as Supplementary Material three full-length multiple sequence alignments of representative sequences and transmembrane predictions for the EXPERA domain-containing families: TM6SF, MAC30/TMEM97 and EBP (Figures S1–S3).

Figure S1 **TM6SF family representative full-length alignment and transmembrane prediction**. The sequence variant identified in TM6SF2 is labeled in red (Holmen et al., 2014; Kozlitina et al., 2014; Sookoian et al., 2014). EXPERA domains limits are marked with red bars (light red for the first repeat and dark red for the second repeat) above the alignment. The most conserved positions of both EXPERA domains (E101 and E255) are labeled. TMHMM helix transmembrane (Krogh et al., 2001) predictions are shown below each input sequence (consensus of these predictions is shown in Figure 3). The alignment was presented with the program Belvu (Sonnhammer and Hollich, 2005) using a coloring scheme indicating the average BLOSUM62 scores (which are correlated with amino acid conservation) of each alignment column: black (>3), gray (between 3 and 1.5) and light gray (between 1.5 and 0.5). Sequences are named according to their UniProt identifications (Wu et al., 2006). Human proteins identifications are underlined in red (TM6SF2, Q9BZW4_HUMAN and TM6SF1, Q9BZW5_HUMAN). Species abbreviations: CALMI, *Callorhynchus milii* (Australian ghost shark); CIOIN, *Ciona intestinalis*; CRAGI, *Crassostrea gigas* (Pacific oyster); HUMAN, *Homo sapiens*; MONBE, *Monosiga brevicollis* (Choanoflagellate); ORENI, *Oreochromis niloticus* (Nile tilapia); SALR5, *Salpingoeca rosetta* (Choanoflagellate); STRPU, *Strongylocentrotus purpuratus* (Purple sea urchin); TETUR, *Tetranychus urticae* (Chelicerata); XENTR, *Xenopus tropicalis*.

Figure S2 **MAC30 family representative full-length alignment and transmembrane prediction**. The extent of the EXPERA domain is marked with a yellow bar above the alignment. The most conserved position of the EXPERA superfamily (D56) is labeled. TMHMM helix transmembrane (Krogh et al., 2001) predictions are shown below each input sequence (consensus of these predictions is shown in Figure 3). The alignment was presented with the program Belvu (Sonnhammer and Hollich, 2005) using a coloring scheme indicating the average BLOSUM62 scores (which are correlated with amino acid conservation) of each alignment column: black (>3), gray (between 3 and 1.5) and light gray (between 1.5 and 0.5). Sequences are named according to their UniProt identifications (Wu et al., 2006). MAC30/TMEM97 human protein identification is underlined in yellow (Q5BJF2_HUMAN). Species abbreviations: 9CHLO, *Coccomyxa subellipsoidea*; ACACA, *Acanthamoeba castellanii*; ARATH, *Arabidopsis thaliana* (Mouse-ear cress); BYSSP, *Byssochlamys spectabilis*; CANAW, *Candida albicans*; CLAL4, *Clavispora lusitaniae*; COCIM, *Coccidioides immitis*; DEBHA, *Debaryomyces hansenii* (Yeast); HUMAN, *Homo sapiens*; LOTGI, *Lottia gigantea* (Giant owl limpet); MARBU, *Marssonina brunnea*; MICGM, *Mycosphaerella graminicola*; MICPC, *Micromonas pusilla*; MUSAM, *Musa acuminata*; PHATC, *Phaeodactylum tricornutum*; PICPG, *Komagataella pastoris* (Yeast); PICSI, *Picea sitchensis*; PICST, *Scheffersomyces stipitis*; PRUPE, *Prunus persica*; PSED2, *Pseudogymnoascus destructans*; SCHPO, *Schizosaccharomyces pombe* (Fission yeast); SELML, *Selaginella moellendorffii* (Spikemoss); TRIAD, *Trichoplax adhaerens*; VOLCA, *Volvox carteri* (Green alga); XENTR, *Xenopus tropicalis*; YEAST, *Saccharomyces cerevisiae* (Baker's yeast).

Figure S3 **EBP family representative full-length alignment and transmembrane prediction**. The extent of the EXPERA domain is marked with a violet bar above the alignment. The most conserved position of the EXPERA superfamily (D108) is labeled. TMHMM helix transmembrane (Krogh et al., 2001) predictions are shown below each input sequence (consensus of these predictions is shown in Figure 3). The alignment was presented with the program Belvu (Sonnhammer and Hollich, 2005) using a coloring scheme indicating the average BLOSUM62 scores (which are correlated with amino acid conservation) of each alignment column: black (>3), gray (between 3 and 1.5) and light gray (between 1.5 and 0.5). Sequences are named according to their UniProt identifications (Wu et al., 2006). Human proteins identifications are underlined in violet (EBP, Q15125_HUMAN and EBPL, Q9BY08_HUMAN). Species abbreviations: 9AGAR, *Moniliophthora roreri*; 9GLOM, *Rhizophagus irregularis*; ACACA, *Acanthamoeba castellanii*; ARATH, *Arabidopsis thaliana* (Mouse-ear cress); ASPO3, *Aspergillus oryzae*; BRAFL, *Branchiostoma floridae* (Amphioxus); CAEEL, *Caenorhabditis elegans*; CAPO3, *Capsaspora owczarzaki*; COPC7, *Coprinopsis cinerea*; CRAGI, *Crassostrea gigas* (Pacific oyster); HUMAN, *Homo sapiens*; LOTGI, *Lottia gigantea* (Giant owl limpet); MAGO7, *Magnaporthe oryzae*; MONDO, *Monodelphis domestica* (opossum); NAEGR, *Naegleria gruberi* (Amoeba); PHACS, *Phanerochaete carnosa*; RHOT1, *Rhodosporidium toruloides*; SELML, *Selaginella moellendorffii* (Spikemoss); STRPU, *Strongylocentrotus purpuratus* (Purple sea urchin); TALSN, *Talaromyces stipitatus*; TRIAD, *Trichoplax adhaerens*; VERA1, *Verticillium alfalfae*.

## Abbreviations

TM6SF2: Transmembrane 6 Superfamily Member 2
TM6SF1: Transmembrane 6 Superfamily Member 1
MAC30: Meningioma-Associated protein 30
TMEM97: Transmembrane Protein 97
EBP: Emopamil Binding Protein
NAFLD: Non-alcoholic Fatty Liver Disease
CDPX2: Chondro-Dysplasia Punctata 2, X-linked dominant
MEND: Male EBP Disorder with Neurological Defects.
