# Supplementary figures and images for: TM6SF2 and MAC30, new enzyme homologs in sterol metabolism and common metabolic disease
Source: Front Genet. 2014 Dec 11;5:439. doi: 10.3389/fgene.2014.00439 (PMC4263179; doi:10.3389/fgene.2014.00439)

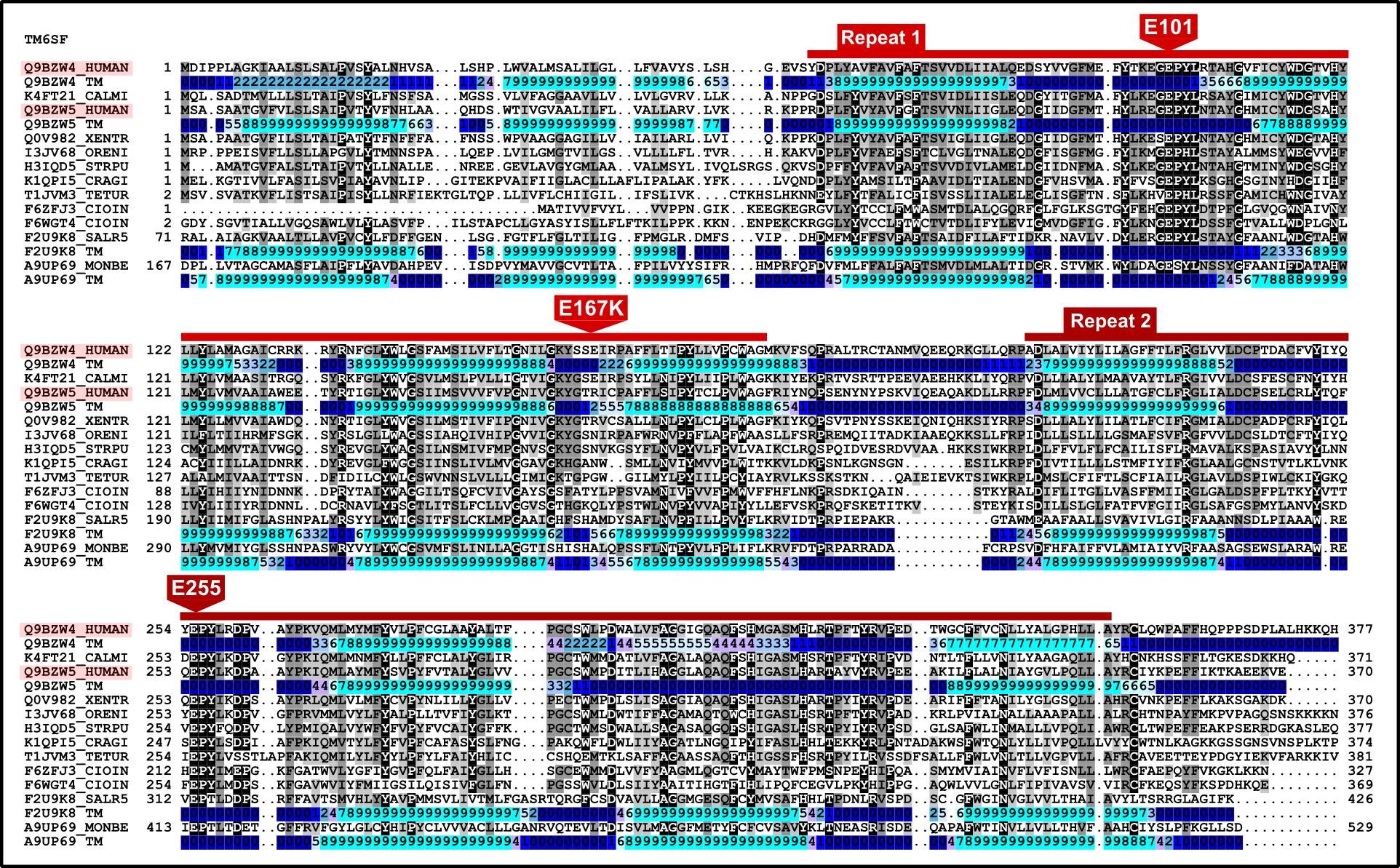

Supplement: Figure S1 — TM6SF family representative full-length alignment and transmembrane prediction. The sequence variant identified in TM6SF2 is labeled in red (Holmen et al., 2014; Kozlitina et al., 2014; Sookoian et al., 2014). EXPERA domains limits are marked with red bars (light red for the first repeat and dark red for the second repeat) above the alignment. The most conserved positions of both EXPERA domains (E101 and E255) are labeled. TMHMM helix transmembrane (Krogh et al., 2001) predictions are shown below each input sequence (consensus of these predictions is shown in Figure 3). The alignment was presented with the program Belvu (Sonnhammer and Hollich, 2005) using a coloring scheme indicating the average BLOSUM62 scores (which are correlated with amino acid conservation) of each alignment column: black (>3), gray (between 3 and 1.5) and light gray (between 1.5 and 0.5). Sequences are named according to their UniProt identifications (Wu et al., 2006). Human proteins identifications are underlined in red (TM6SF2, Q9BZW4_HUMAN and TM6SF1, Q9BZW5_HUMAN). Species abbreviations: CALMI, Callorhynchus milii (Australian ghost shark); CIOIN, Ciona intestinalis; CRAGI, Crassostrea gigas (Pacific oyster); HUMAN, Homo sapiens; MONBE, Monosiga brevicollis (Choanoflagellate); ORENI, Oreochromis niloticus (Nile tilapia); SALR5, Salpingoeca rosetta (Choanoflagellate); STRPU, Strongylocentrotus purpuratus (Purple sea urchin); TETUR, Tetranychus urticae (Chelicerata); XENTR, Xenopus tropicalis. [file Image1.JPEG]

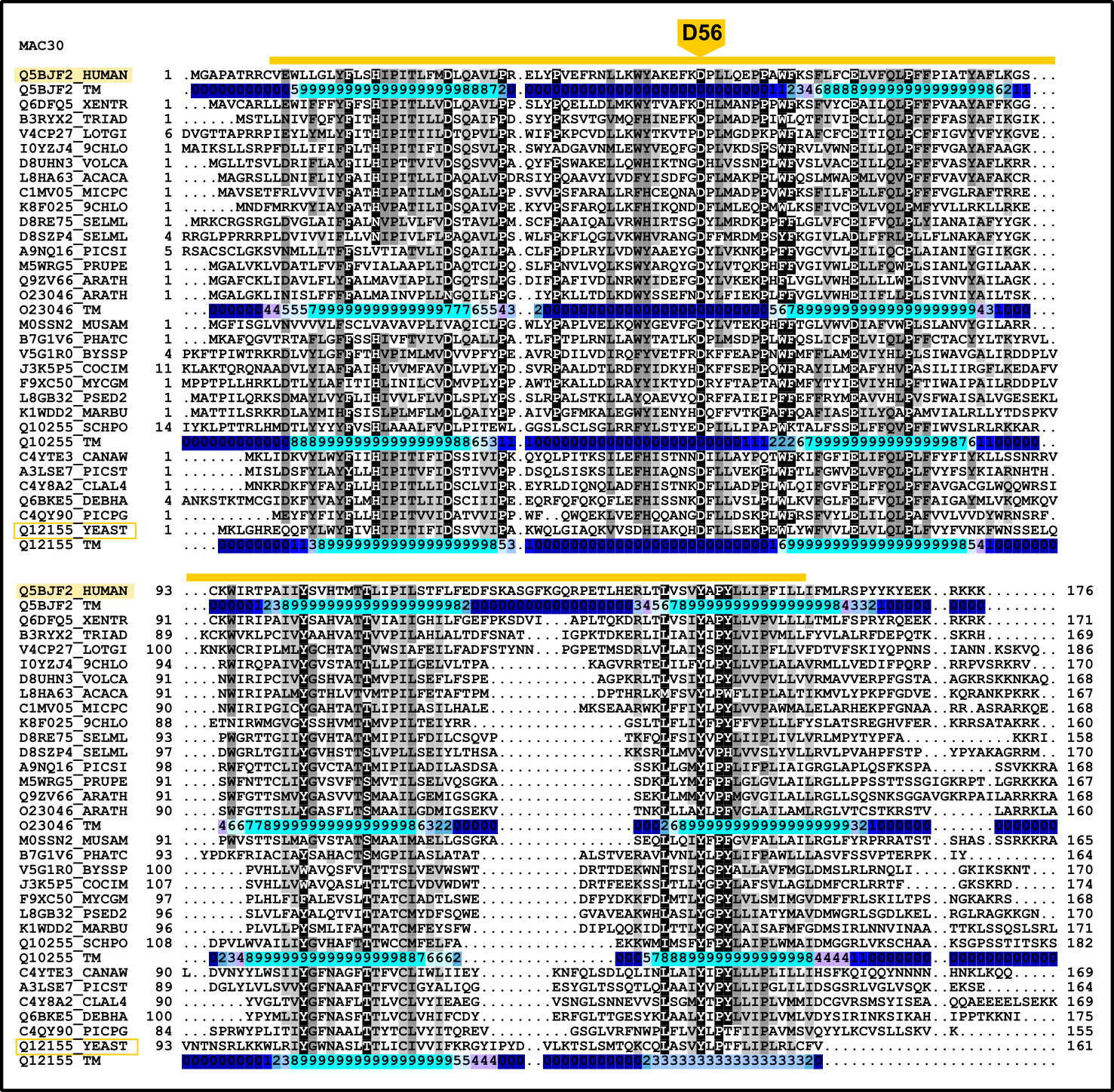

Supplement: Figure S2 — MAC30 family representative full-length alignment and transmembrane prediction. The extent of the EXPERA domain is marked with a yellow bar above the alignment. The most conserved position of the EXPERA superfamily (D56) is labeled. TMHMM helix transmembrane (Krogh et al., 2001) predictions are shown below each input sequence (consensus of these predictions is shown in Figure 3). The alignment was presented with the program Belvu (Sonnhammer and Hollich, 2005) using a coloring scheme indicating the average BLOSUM62 scores (which are correlated with amino acid conservation) of each alignment column: black (>3), gray (between 3 and 1.5) and light gray (between 1.5 and 0.5). Sequences are named according to their UniProt identifications (Wu et al., 2006). MAC30/TMEM97 human protein identification is underlined in yellow (Q5BJF2_HUMAN). Species abbreviations: 9CHLO, Coccomyxa subellipsoidea; ACACA, Acanthamoeba castellanii; ARATH, Arabidopsis thaliana (Mouse-ear cress); BYSSP, Byssochlamys spectabilis; CANAW, Candida albicans; CLAL4, Clavispora lusitaniae; COCIM, Coccidioides immitis; DEBHA, Debaryomyces hansenii (Yeast); HUMAN, Homo sapiens; LOTGI, Lottia gigantea (Giant owl limpet); MARBU, Marssonina brunnea; MICGM, Mycosphaerella graminicola; MICPC, Micromonas pusilla; MUSAM, Musa acuminata; PHATC, Phaeodactylum tricornutum; PICPG, Komagataella pastoris (Yeast); PICSI, Picea sitchensis; PICST, Scheffersomyces stipitis; PRUPE, Prunus persica; PSED2, Pseudogymnoascus destructans; SCHPO, Schizosaccharomyces pombe (Fission yeast); SELML, Selaginella moellendorffii (Spikemoss); TRIAD, Trichoplax adhaerens; VOLCA, Volvox carteri (Green alga); XENTR, Xenopus tropicalis; YEAST, Saccharomyces cerevisiae (Baker's yeast). [file Image2.JPEG]

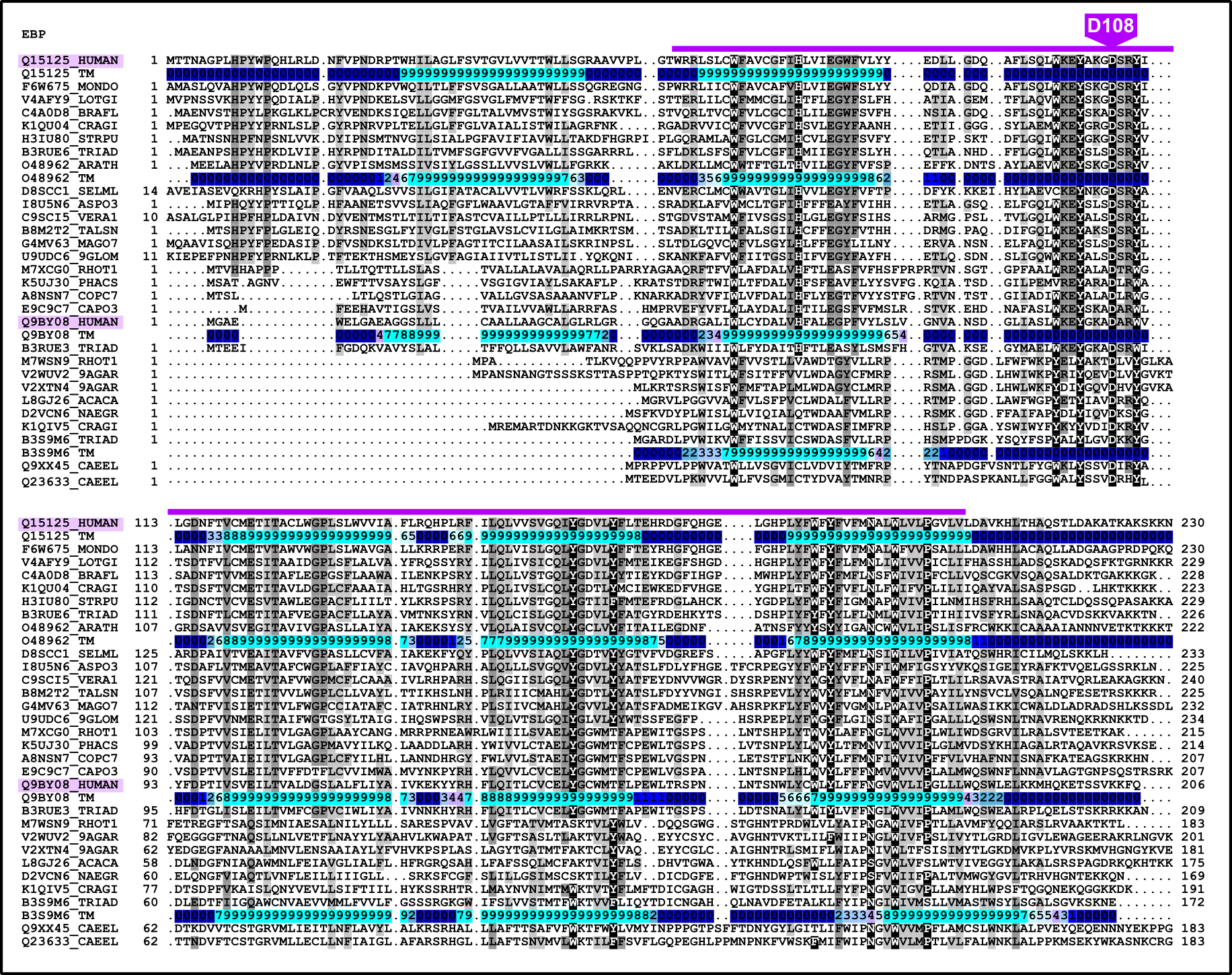

Supplement: Figure S3 — EBP family representative full-length alignment and transmembrane prediction. The extent of the EXPERA domain is marked with a violet bar above the alignment. The most conserved position of the EXPERA superfamily (D108) is labeled. TMHMM helix transmembrane (Krogh et al., 2001) predictions are shown below each input sequence (consensus of these predictions is shown in Figure 3). The alignment was presented with the program Belvu (Sonnhammer and Hollich, 2005) using a coloring scheme indicating the average BLOSUM62 scores (which are correlated with amino acid conservation) of each alignment column: black (>3), gray (between 3 and 1.5) and light gray (between 1.5 and 0.5). Sequences are named according to their UniProt identifications (Wu et al., 2006). Human proteins identifications are underlined in violet (EBP, Q15125_HUMAN and EBPL, Q9BY08_HUMAN). Species abbreviations: 9AGAR, Moniliophthora roreri; 9GLOM, Rhizophagus irregularis; ACACA, Acanthamoeba castellanii; ARATH, Arabidopsis thaliana (Mouse-ear cress); ASPO3, Aspergillus oryzae; BRAFL, Branchiostoma floridae (Amphioxus); CAEEL, Caenorhabditis elegans; CAPO3, Capsaspora owczarzaki; COPC7, Coprinopsis cinerea; CRAGI, Crassostrea gigas (Pacific oyster); HUMAN, Homo sapiens; LOTGI, Lottia gigantea (Giant owl limpet); MAGO7, Magnaporthe oryzae; MONDO, Monodelphis domestica (opossum); NAEGR, Naegleria gruberi (Amoeba); PHACS, Phanerochaete carnosa; RHOT1, Rhodosporidium toruloides; SELML, Selaginella moellendorffii (Spikemoss); STRPU, Strongylocentrotus purpuratus (Purple sea urchin); TALSN, Talaromyces stipitatus; TRIAD, Trichoplax adhaerens; VERA1, Verticillium alfalfae. [file Image3.JPEG]
